# Genetic variants associated with metabolic dysfunction‐associated fatty liver disease in western China

**DOI:** 10.1002/jcla.24626

**Published:** 2022-07-26

**Authors:** Shenling Liao, Kang An, Zhi Liu, He He, Zhenmei An, Qiaoli Su, Shuangqing Li

**Affiliations:** ^1^ Department of Laboratory Medicine West China Hospital, Sichuan University Chengdu China; ^2^ Department of General Practice West China Hospital, Sichuan University Chengdu China; ^3^ Department of Endocrinology and Metabolism West China Hospital, Sichuan University Chengdu China

**Keywords:** *GATAD2A*, *GCKR*, *MBOAT7*, metabolic dysfunction‐associated fatty liver disease, *PNPLA3*, *TM6SF2*

## Abstract

**Introduction:**

We aimed to confirm the association between some single nucleotide polymorphisms (SNPs) and metabolic dysfunction‐associated fatty liver disease (MAFLD) in western China.

**Methods:**

A total of 286 cases and 250 healthy controls were enrolled in our study. All samples were genotyped for patatin‐like phospholipase domain containing 3 (*PNPLA3*) rs738409, transmembrane 6 superfamily member 2 (*TM6SF2*) rs58542926, membrane‐bound O‐acyltransferase domain containing 7 (*MBOAT7*) rs641738, glucokinase regulator (*GCKR*) rs1260326 and rs780094, and GATA zinc finger domain containing 2A (*GATAD2A*) rs4808199. Using logistic regression analysis, we evaluated the association between MAFLD and each SNP under different models. Multiple linear regression was used to find the association between SNPs and laboratory characteristics. Multifactor dimensionality reduction was applied to test SNP–SNP interactions.

**Results:**

The recessive model and additive model of *PNPLA3* rs738409 variant were related to MAFLD (odds ratio [OR] = 1.791 and 1.377, respectively, *p* = 0.038 and 0.027, respectively). However, after Benjamini‐Hochberg adjustment for multiple tests, all associations were no longer statistically significant. *PNPLA3* rs738409 correlated with AST levels. *GCKR* rs780094 and rs1260326 negatively correlated with serum glucose but positively correlated with triglycerides in MAFLD. Based on MDR analysis, the best single‐locus and multilocus models for MAFLD risk were rs738409 and six‐locus models, respectively.

**Conclusions:**

In the Han population in western China, no association was found between these SNPs and the risk of MAFLD. *PNPLA3* rs738409 was associated with aspartate aminotransferase levels in MAFLD patients. *GCKR* variants were associated with increased triglyceride levels and reduced serum fasting glucose in patients with MAFLD.

## Graphical abstract

The study aimed to confirm the association between some single nucleotide polymorphisms (SNPs) and metabolic dysfunction‐associated fatty liver disease (MAFLD) in western China. We found no association between these SNPs and the risk of MAFLD after Benjamini‐Hochberg adjustment. PNPLA3 rs738409 was associated with the AST levels in MAFLD patients. GCKR variants were associated with increased triglycerides levels and reduced serum fasting glucose in patients with MAFLD. In addition, there was no synergistic SNP–SNP interaction between PNPLA3 rs738409 and other SNPs on MAFLD.

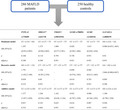

## 1 INTRODUCTION

Metabolic dysfunction‐associated fatty liver disease (MAFLD), formerly named nonalcoholic associated fatty liver disease (NAFLD), is increasing worldwide, and the global prevalence is 29.8% (95% confidence interval: 28.6%–31.1%).^1^ MAFLD has become the most common chronic liver disease, characterized by steatosis and metabolic dysfunction. The diagnosis of MAFLD has changed from previous exclusionary diagnostic criteria to current positive criteria.^2^ Studies using the diagnostic criteria for NAFLD and MAFLD separately in the same population reported that the updated definition of MAFLD did not significantly change the prevalence compared with NAFLD.^3^, ^4^ MAFLD is becoming a global economic burden and is a growing strain on healthcare systems for disease management.^5^

There is growing evidence that genetics and environmental factors play fundamental roles in the development and progression of MAFLD.^6^ The heritability of MAFLD has been confirmed with evidence from data from epidemiological, familial aggregation, and twin studies, with heritability estimates ranging from 20% to 70%.^7^, ^8^, ^9^, ^10^ With the increasing number of studies on MAFLD susceptibility, progression based on genome‐wide association studies and large candidate gene studies,^11^, ^12^, ^13^ several single nucleotide polymorphisms (SNPs) have significantly contributed to the development of MAFLD, such as patatin‐like phospholipase domain containing 3 (*PNPLA3*) rs738409,^11^, ^12^ transmembrane 6 superfamily member 2 (*TM6SF2*) rs58542926,^14^, ^15^ membrane‐bound O‐acyltransferase domain containing 7 (*MBOAT7*) rs641738,^16^ glucokinase regulator (*GCKR*) rs1260326 and rs780094,^17^ and GATA zinc finger domain containing 2A (*GATAD2A*) rs4808199.^18^

*PNPLA3* rs738409 encodes *PNPLA3* I148 M, which is a robust variant associated with MAFLD in multiple ethnicities.^19^, ^20^ It is currently considered that *PNPLA3* I148 M promotes hepatic steatosis via lipid reprogramming characterized by triglyceride accumulation, polyunsaturated fatty acid depletion, and signature of transducer and activator of transcription 3 (STAT3) activation leading to inflammatory pathway activation.^21^, ^22^ *TM6SF2* rs58542926 likely results in functional loss, VLDL secretion impairment, and hepatic steatosis fibrosis promotion.^23^ *MBOAT7* rs641738 is associated with higher triglyceride synthesis by a noncanonical phosphatidylinositol pathway.^24^ *GCKR* rs1260326 and rs780094 regulate glucokinase and likely provide more substrates for lipogenesis.^25^ *GATAD2A* rs4808199 is an intron variant that was reported to be associated with MAFLD in Japanese individuals,^18^ but there have been few studies about the potential mechanism.

There is large interethnic variability in an individual's susceptibility to MAFLD.^26^, ^27^, ^28^ The association between *PNPLA3* rs738409 and MAFLD is robust. However, the association between *TM6SF2* rs58542926, *GCKR* rs1260326, and rs780094 and MAFLD in the Chinese population is not consistent with Europeans. *MBOAT7* rs641738 and *GATAD2A* rs4808199 lack investigation in the Chinese population. Therefore, we selected these six SNPs to explore the relationship between MAFLD in western China and investigate the association between SNPs and laboratory characteristics in MAFLD. Multifactor dimensionality reduction (MDR) is a wide method to analyze gene–gene interactions, which aims to reduce the complexity of multilocus information by pooling genotypes into high‐risk and low‐risk groups, thus reducing them to a one‐dimensional variable.^29^ Additionally, we attempted to find SNP–SNP interactions in MAFLD using MDR analysis.

## 2 MATERIALS AND METHODS

### 2.1 Subjects and data collection

We enrolled 286 cases and 250 healthy controls. Subjects were recruited from West China Hospital of Sichuan University from December 2019 to December 2020. All subjects were unrelated and ethnically Han Chinese. In terms of the diagnostic criteria of MAFLD, the international expert consensus statement states that, besides the evidence of hepatic steatosis, it also meets one of the following three criteria: overweight/obesity, type 2 diabetes, or evidence of metabolic dysregulation.^2^ In the study, hepatic steatosis was supported by imaging evidence, such as abdominal ultrasonography, computed tomography, magnetic resonance imaging, or controlled attenuation parameter score. Overweight/obesity was defined as body mass index (BMI) ≥23 kg/m^2^ for Asians. Metabolic dysregulation was characterized by high blood pressure, high triglycerides, low HDL‐cholesterol, prediabetes, or receiving specific drug treatment for these metabolic disorders. Controls consisted of individuals from the health management department of the hospital without hepatic steatosis on imaging, and the sex and age were matched. All subjects with viral infections, drug‐induced liver injury, autoimmune hepatitis, total parenteral nutrition, or tumors were excluded, regardless of group. Our study was approved by the institutional ethics committee, and informed consent was obtained from all participants.

The data, including age, sex, BMI, medical history, and routine biochemical indexes, were obtained from the laboratory information system and hospital information system. Routine biochemical indexes included total bilirubin, direct bilirubin, indirect bilirubin, albumin, globulin, alanine transaminase (ALT), aspartate aminotransferase (AST), glucose, urea, creatinine, serum uric acid, triglyceride (TG), cholesterol, low‐density lipoprotein cholesterol (LDL‐C), high‐density lipoprotein cholesterol (HDL‐C), alkaline phosphatase (ALP), and gamma‐glutamyl transpeptidase (GGT).

### 2.2 Specimen collection and SNP genotyping

For genomic DNA extraction, two milliliters of whole blood samples were collected with EDTA anticoagulant. The magnetic bead method was used to extract DNA according to the manufacturer's instructions (Danfeng Technology, Chengdu, China). Genotyping was performed using the multiplex polymerase chain reaction (PCR) and multiplex ligase detection reaction (LDR) method. After concentration and purity measurements, 50 ng DNA template was used for PCR. Regarding quality control, 10% of samples were selected randomly and genotyped twice to ensure accuracy. Negative control was used for contamination detection and electrophoresis on the PCR products to observe whether the reaction was successful. The PCR primers were shown in Table S1, and PCR was carried out with the initial denaturation at 95°C for 2 min, 40 cycles for denaturation at 95°C for 30 s, annealing at 56°C for 30 s and extension at 72°C for 60 s, and a final extension at 72°C for 10 min. Information on the LDR reaction probe was shown in Table S2, and the LDR program was 95°C for 2 min and 40 cycles at 94°C for 15 s and 50°C for 25 s. The products were sequenced by PRISM 3730 (ABI, USA). All samples were genotyped for *PNPLA3* rs738409, *MBOAT7* rs641738, *GATAD2A* rs4808199, *GCKR* rs1260326, *GCKR* rs780094, and *TM6SF2* rs58542926. HaploReg v4.1 (https://pubs.broadinstitute.org/mammals/haploreg/haploreg.php) was used to annotate the potential functional role of these SNPs^30^ (Table 1).

**TABLE 1:** Basic information of SNPs, including the frequencies of SNP genotypes, the *p* value of the Hardy–Weinberg equilibrium equation, and bioinformatics function annotations

SNP	Genotypes	Control group (*n*)	*p*	Case group (*n*)	*p*	HaploReg
*PNPLA3* rs738409 C > G	CC	105	0.912	91	0.732	Enhancer histone marks, Motifs changed, NHGRI/EBI GWAS hits, selected eQTL hits
	CG	114		137		
	GG	30		56		
*TM6SF2* rs58542926 C > T	CC	221	0.401	239	0.194	Enhancer histone marks, Motifs changed, selected eQTL hits
	CT	25		35		
	TT	0		3		
*MBOAT7* rs641738 C > T	CC	136	0.468	148	0.826	Promoter histone marks, enhancer histone marks, DNAse, proteins bound, Motifs changed, GRASP QTL hits, selected eQTL hits
	CT	93		113		
	TT	20		23		
*GCKR* rs780094 C > T	CC	55	0.717	63	0.957	Enhancer histone marks, DNAse, proteins bound, Motifs changed, HGRI/EBI GWAS hits, GRASP QTL hits, selected eQTL hits
	CT	121		142		
	TT	73		79		
*GCKR* rs1260326 C > T	CC	54	0.520	59	0.954	Enhancer histone marks, Motifs changed, HGRI/EBI GWAS hits, GRASP QTL hits, selected eQTL hits
	CT	118		139		
	TT	76		83		
*GATAD2A* rs4808199 G > A	GG	153	0.852	171	0.051	Promoter histone marks, enhancer histone marks, DNAse, Motifs changed, GRASP QTL hits, selected eQTL hits
	GA	85		91		
	AA	11		22		

### 2.3 Statistical analysis

We used the Genetic Association Study (GAS) Power Calculator (http://csg.sph.umich.edu/abecasis/CaTS/gas_power_calculator/index.html) to calculate the sample size. For the parameter, the prevalence of MAFLD was approximately 30%, the disease allele frequency was the average variant allele frequency of our 6 studied SNPs (0.39), and the significance level was 0.008. The results showed that 250 cases (case: control = 1:1) could provide enough power (>0.8) to detect moderate genetic effects (genotype relative risk >1.4). Statistical analysis was performed using IBM SPSS 21.0 statistical software (USA) and R software (version 4.0.5, Austria). The allele frequencies of all SNP genotypes obeyed the Hardy–Weinberg equilibrium (HWE) equation (*p* > 0.05, Table 1). The mean and standard deviation for normal data were presented, and the median and interquartile range for nonnormal data. The chi‐square test was conducted on sex variables, and the Student's *t* test and the Mann–Whitney U test were performed for quantitative variables. Using logistic regression analysis, we evaluated the risk of MAFLD associated with each SNP under the dominant model, the recessive model, and the additive model after inputting age, sex, and BMI for adjustment. Then, we used multiple linear regression to look for the association between SNPs and laboratory indexes after inputting age, sex, and BMI for adjustment. SNP–SNP interactions were tested using MDR software version 3.0.2 (https://sourceforge.net/projects/mdr/).^31^ Benjamini–Hochberg adjusted *p* values ≤0.05 were considered statistically significant.

## 3 RESULTS

### 3.1 Study population

In total, 286 cases and 250 healthy controls were screened. The characteristics of the study populations are shown in Table 2. There was no significant difference in the distribution of sex between MAFLD patients and controls. Globin, urea, creatine, cholesterol, low‐density lipoprotein cholesterol (LDL‐C), and alkaline phosphatase (ALP) were not significantly different between the two groups. Patients with MAFLD presented a higher BMI, elevated glucose, triglycerides (TGs), and serum uric acid levels.

**TABLE 2:** Characteristics of the study populations. Data are presented as the median and interquartile range or mean and standard deviation, and the Mann–Whitney *U* test or Student's *t* test was performed for quantitative variables

Variables	Control	MAFLD	*p*
Sex (male%)	73.2%	75.0%	0.637
Age	49.00 (17.00)	51.00 (18.00)	0.075
BMI	23.95 (3.57)	27.12 (4.67)	<0.001
Total bilirubin (μmol/L)	13.70 (8.62)	15.84 (7.71)	<0.001
Direct bilirubin (μmol/L)	4.40 (3.07)	4.86 (2.27)	<0.001
Indirect bilirubin (μmol/L)	9.32 (6.20)	10.90 (5.34)	0.002
Albumin (g/L)	47.40 (7.50)	49.05 (4.23)	<0.001
Globulin (g/L)^a^	27.57 (3.26)	27.45 (4.87)	0.739
ALT (IU/L)	20.00 (14.00)	29.00 (29.00)	<0.001
AST (IU/L)	18.00 (6.00)	21.00 (14.00)	<0.001
Glucose (mmol/L)	5.47 (0.88)	6.13 (3.16)	<0.001
Urea (mmol/L)	4.91 (2.13)	4.86 (1.60)	0.830
Creatinine (μmol/L)^a^	77.56 (17.35)	75.77 (15.06)	0.211
Serum uric acid (μmol/L)	316.00 (110.00)	350.50 (117.25)	<0.001
TG (mmol/L)	1.29 (1.00)	2.03 (1.43)	<0.001
CHOL (mmol/L)^a^	4.74 (1.05)	4.77 (1.16)	0.781
HDL‐C (mmol/L)	1.35 (0.50)	1.08 (0.44)	<0.001
LDL‐C (mmol/L)^a^	2.97 (0.84)	3.06 (0.93)	0.218
ALP (IU/L)	75.00 (27.00)	75.00 (29.00)	0.564
GGT (IU/L)	26.00 (22.50)	36.00 (36.00)	<0.001

Abbreviations: ALP, alkaline phosphatase; ALT, alanine transaminase; AST, aspartate aminotransferase; CHOL, cholesterol; GGT, gamma‐glutamyl transpeptidase; HDL‐C, high‐density lipoprotein cholesterol; LDL‐C, low‐density lipoprotein cholesterol; TG, triglycerides.

^a^ Normal data are presented as the mean ± standard deviation, and Student's *t* test was used to compare continuous variables.

### 3.2 Association between genotypes and MAFLD

Six SNPs were genotyped, and the genotype distribution in MAFLD and controls was shown in Table 1. Logistic regression analysis was performed to evaluate the association between gene variants and MAFLD after adjustment for age, sex, and BMI. The dominant model, recessive model, and additive model for each SNP were analyzed, and the results are shown in Table 3.

**TABLE 3:** Association between the genetic model for each SNP and MAFLD. Logistic regression analysis was performed to evaluate the association between gene variants and MAFLD after adjustment for age, sex, and BMI

	*PNPLA3* rs738409	*MBOAT7* rs641738	*TM6SF2* rs58542926	*GCKR* rs780094	*GCKR* rs1260326	*GATAD2A* rs4808199
Dominant model	CC vs. CG + GG	CC vs. CT + TT	CC vs. CT + TT	CC vs. CT + TT	CC vs. CT + TT	GG vs. GA + AA
OR (95% CI)	1.397 (0.933, 2.093)	1.375 (0.926, 2.043)	1.088 (0.577, 2.501)	0.929 (0.582, 1.484)	1.015 (0.630, 1.636)	0.980 (0.655, 1.465)
*p*	0.104	0.114	0.795	0.758	0.951	0.92
adjust *p* ^a^	0.388	0.388	0.951	0.951	0.951	0.951
Recessive model	CC + CG vs. GG	CT + CC vs. TT	CT + CC vs. TT	CT + CC vs. TT	CT + CC vs. TT	GA + GG vs. AA
OR (95% CI)	1.791 (1.032, 3.108)	1.156 (0.567, 2.354)	—	0.894 (0.578, 1.382)	0.942 (0.613, 1.445)	2.066 (0.880, 4.847)
*p*	0.038	0.69	—	0.614	0.783	0.095
adjust *p*	0.323	0.951	—	0.951	0.951	0.388
Additive model	CC vs. CG vs. GG	CC vs. CT vs. TT	CC vs. CT vs. TT	CC vs. CT vs. TT	CC vs. CT vs. TT	GG vs. GA vs. AA
OR (95% CI)	1.377 (1.037, 1.828)	1.244 (0.916, 1.690)	1.162 (0.636, 2.126)	0.932 (0.707, 1.228)	0.980 (0.744, 1292)	1.100 (0.796, 1.520)
*p*	0.027	0.163	0.625	0.616	0.887	0.564
adjust *p*	0.323	0.462	0.951	0.951	0.951	0.951

^a^ The adjusted *p* value was adjusted by Benjamini–Hochberg.

The recessive model and additive model of the *PNPLA3* rs738409 variant were potentially related to MAFLD (odds ratio [OR] = 1.791 and 1.377, respectively, *p* = 0.038 and 0.027, respectively). However, after Benjamini–Hochberg adjustment for multiple tests, all associations were no longer statistically significant. As for *TM6SF2* rs58542926 genotype TT, only three patients were identified, so the recessive model could not be analyzed. Other gene variants were not statistically correlated with MAFLD.

### 3.3 Association between genotypes and quantitative traits in MAFLD

Multiple linear regression was performed to evaluate the association between quantitative traits in the MAFLD group and genetic models after adjustment for age, sex, and BMI. As shown in Table 4, *PNPLA3* rs738409 correlated with AST levels. The T allele of *GCKR* rs1260326 and the T allele of *GCKR* rs780094 negatively correlated with serum glucose. However, the T allele of *GCKR* rs1260326 and the T allele of *GCKR* rs780094 positively correlated with triglycerides. In addition, there were no significant associations between other quantitative traits in MAFLD and gene variants.

**TABLE 4:** The association between quantitative traits in the MAFLD group and genetic models after adjustment for age, sex, and BMI

Traits	SNP	B (95% CI)	*p*	Adjust *p* ^a^
TG	*GCKR* rs1260326 recessive model	0.649 (0.125, 1.173)	0.015	0.022
TG	*GCKR* rs780094 recessive model	0.706 (0.178, 1.234)	0.009	0.022
GLU	*GCKR* rs1260326 dominant model	−1.217 (−2.004, −0.390)	0.004	0.022
GLU	*GCKR* rs1260326 additive model	−0.618 (−1.096, −0.140)	0.011	0.022
GLU	*GCKR* rs780094 dominant model	−0.892 (−1.699, −0.084)	0.031	0.031
AST	*PNPLA3* rs738409 recessive model	13.774 (2.404, 25.145)	0.018	0.022

^a^ The adjusted *p* value was adjusted by Benjamini–Hochberg.

### 3.4 The impact of SNP–SNP interactions on MAFLD

**FIGURE 1:**
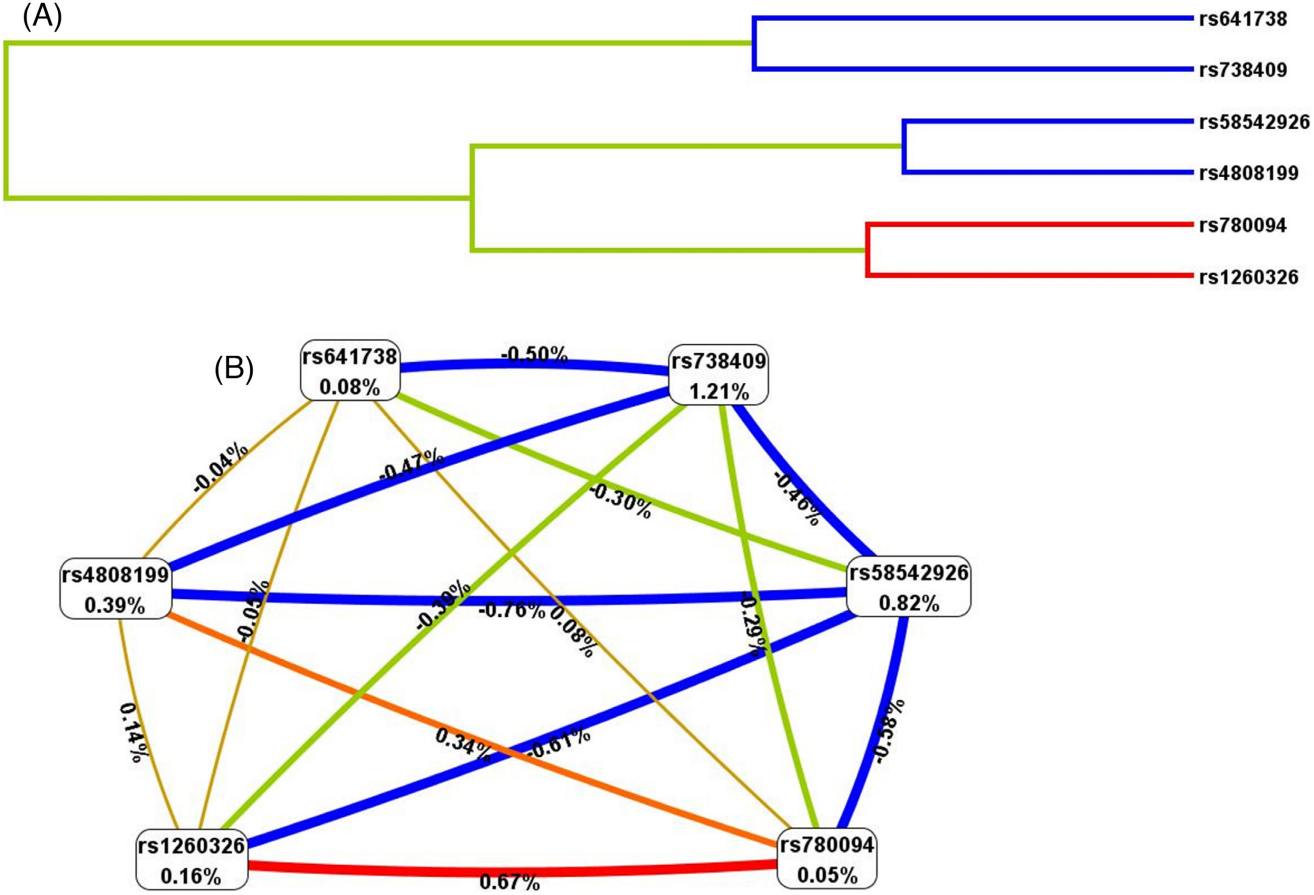
The entropy of SNP–SNP interactions in the dendrogram (A) and circle graph (B). (A) Short lines represented stronger interactions, and long lines represented weaker interactions. (B) Entropy values of the SNPs were presented in the cells, showing the main independent effects. The connecting lines indicated pairwise interactive effects. Red and blue represented synergism, and redundancy, respectively. Negative percent entropy indicates redundancy, while positive percent entropy indicates synergism

The results of the optimal SNP model with different numbers of loci were displayed in Table 5.^31^ As shown in the table, the single‐locus model (*PNPLA3* rs738409) had the best testing balance accuracy (0.533), and the cross‐validation consistency was 10/10, making it the best single‐site model for the risk of MAFLD. Additionally, the six‐locus SNP model achieved good testing balance accuracy (0.511) and cross‐validation consistency (10/10). The entropy of SNP–SNP interactions were shown in Figure 1. MDR used entropy measurements to evaluate and visualize the interactions between variable combinations. The blue line and negative numbers indicated that there was likely redundantly in regulating the risk of MAFLD. The dendrogram (A) and circle graph (B) both showed that *PNPLA3* rs738409 redundantly interacted with other SNPs. Most SNP–SNP interactions were negligible or neutral, except for the positive interaction effects of *GCKR* rs1260326 and *GCKR* rs780094. However, the positive interaction might be caused by linkage disequilibrium (*R* ^2^ > 0.8). In addition, SNP–SNP interactions evaluated by logistic regression analysis did not find statistically significant associations after Benjamini–Hochberg adjustment (Table S3).

**TABLE 5:** Summary of SNP–SNP interaction models detected by MDR

Model	Training balance accuracy	Testing balance accuracy	Cross‐validation consistency	*p*
rs738409	0.5512	0.5337	10/10	0.0146
rs58542926, rs738409	0.5660	0.5008	5/10	0.0007
rs1260326, rs4808199, rs738409	0.5960	0.4916	5/10	<0.0001
rs1260326, rs4808199, rs641738, rs738409	0.6428	0.5188	9/10	<0.0001
rs58542926, rs1260326, rs4808199, rs641738, rs738409	0.6677	0.5165	8/10	<0.0001
rs58542926, rs780094, rs1260326, rs4808199, rs641738, rs738409	0.6795	0.5105	10/10	<0.0001

## 4 DISCUSSION

MAFLD is a complex metabolic disorder in which the environment and susceptible genes interact. In mainland China, the prevalence of MAFLD varies from 19.18% to 36.41% in regions and provinces, and the prevalence is higher in ethnic minorities.^28^ In our study, we assessed the association between SNPs and MAFLD and the effects of SNP–SNP interactions on MAFLD in the western Chinese Han population. Further, we found that SNPs were related to some quantitative traits in MAFLD.

In our study, the *PNPLA3* G allele was the potential gene variant correlated with MAFLD, which was to be expected. However, possibly due to insufficient sample size, it was not statistically significant after correcting the *p*‐value. *PNPLA3* rs738409 is a recognized genetic factor associated with MAFLD in various populations^13^, ^32^, ^33^, ^34^ and has been confirmed to be associated with the full spectrum of MAFLD.^11^ *PNPLA3* rs738409 was associated with aspartate aminotransferase (AST) levels, which was consistent with Luca Valenti et al.’s study.^35^ Patients with *PNPLA3* rs738409 genotype GG had a higher risk of liver damage. The *PNPLA3* rs738409 variant was associated with triglyceride accumulation within the liver^36^ and was related to lower free cholesterol and triglycerides.^37^ In our study, the *PNPLA3* rs738409 dominant model was not significantly associated with serum triglyceride concentrations (OR: −0.487, 95% CI: −0.990, 0.017, *p*: 0.058). This was consistent with Pirazzi et al., who reported that *PNPLA3* rs738409 was associated with reduced plasma TG levels in very obese individuals.^38^

The *TM6SF2* rs58542926 variant was also associated with a reduction in lipids in most VLDL.^37^, ^39^ *PNPLA3* rs738409 and *TM6SF2* rs58542926 both contributed to the reduced serum triglyceride levels and the decreased risk of cardiovascular disease.^40^, ^41^ However, no association of *TM6SF2* rs58542926 with MAFLD and lipid metabolism indexes was observed in the present study, probably because the frequency of the *TM6SF2* rs58542926 minor allele was too low. The minor allele (T) frequency of *TM6SF2* rs58542926 in different studies was highly variable in the Chinese population,^42^, ^43^ so the association with MAFLD is controversial in the Chinese population.

*GCKR* encodes glucokinase regulatory protein regulating the activity of glucokinase and is involved in de novo lipogenesis.^44^ GCKR variants were related to glucose levels, insulin resistance, and type 2 diabetes.^15^ And diabetes and fatty liver are often associated.^45^ Some studies have identified that *GCKR* rs1260326 and *GCKR* rs780094 were significantly associated with MAFLD.^11^, ^46^ However, there was no correlation between the *GCKR* variants and the risk of MAFLD in the study. On the other hand, *GCKR* rs1260326 and *GCKR* rs780094 were associated with increased TG levels and reduced serum fasting glucose, which was also found in some studies.^47^, ^48^ It was redundant with *PNPLA3* rs738409 in the SNP–SNP interaction, as *PNPLA3* rs738409 was associated with reduced serum TG concentration.

The *MBOAT7* rs641738 variant was associated with reduced *MBOAT7* mRNA expression in hepatocytes, and the deletion of *MBOAT7* increased the accumulation of saturated triglycerides and enhanced lipogenesis.^49^ *MBOAT7* rs641738 was associated with increased hepatic fat, elevated alanine transaminase (ALT) levels, and MAFLD severity in Caucasians, Hispanics, and African American ethnicities, whereas no consistent effect was observed in Asian populations.^16^, ^50^ Consistently, in the Chinese Han population, the association between *MBOAT7* rs641738 and MAFLD was not observed, and the *MBOAT7* rs641738 dominant model was not significantly associated with ALT (OR: 8.739, 95% CI: −0.618, 18.097, *p*: 0.067). We considered that *MBOAT7* rs641738 was likely influenced by ethnicity in the development of MAFLD.

There have been few studies on the association of *GATAD2A* rs4808199 and MAFLD. Takahisa et al. and Arun Kumar et al. found that *GATAD2A* rs4808199 was significantly associated with MAFLD in genome‐wide association studies in the Japanese population.^18^, ^51^ In our study, a significant association of *GATAD2A* rs4808199 with MAFLD was not observed.

MDR is a nonparametric statistical tool for detecting gene–gene interactions or gene–environment interactions. With the employment of MDR analysis, we found that *PNPLA3* rs738409 was a significant single‐locus model and was the best significant predictive model. *TM6SF2* rs58542926 and *PNPLA3* rs738409 were both associated with reduced serum triglyceride concentrations and a lower risk of cardiovascular disease,^40^, ^41^, ^52^ and there was probably a synergistic interaction. Min Xu et al. found that there was an interaction between the *PNPLA3* I148 M and *TMS6F2* E167K variants in MAFLD, and patients with both variants had the highest odds ratio after adjusting for age, sex, and BMI.^34^ However, the present study observed a redundant interaction, likely due to fewer *TM6SF2* rs58542926 CT or TT genotypes in populations. Rs1260326 and rs780094 are *GCKR* variants; a synergistic interaction was observed likely due to linkage disequilibrium.

Our study confirmed that the *PNPLA3* rs738409 G allele was associated with liver damage in the Han population in western China. We found that there was a redundant interaction between *PNPLA3* rs738409 and other SNPs, and *PNPLA3* rs738409 was probably an independent risk factor among genetic factors. Meanwhile, there was no association between the other studied SNPs and MAFLD except that *GCKR* variants were associated with increased TG levels and reduced serum fasting glucose in patients with MAFLD.

As the number of known genetic variants associated with MAFLD increases, MAFLD relevant SNPs based on region and ethnicity could provide more accurate measures of MAFLD genetic susceptibility and possibly predict disease progression and regression. The construction of polygenic scores will help reclassify the disease to reflect functional consequences and thus improve clinical management, moreover, in addition to preventing and treating disease, as well as predicting adverse reactions to drugs.^7^

There were some limitations in our study. First, the candidate SNPs were selected based on literature, there is probable subjective selection bias. Though we did not find the studied SNPs associated with MAFLD, there are other SNPs of the genes studied that may be associated, as well as other genes associated with MAFLD. Samples were not large enough because some significant associations were not observed. Patients from the hospital in Sichuan Province and the results might not represent all of western China. In the control group, subclinical MAFLD was probably included, which may cause bias in the study. In addition, MAFLD patients were accompanied with metabolic dysfunction, some SNPs might directly be associated with the metabolic factors, which are confounding factors for our study.

## 5 CONCLUSIONS

In conclusion, no association was found between these SNPs and the risk of MAFLD in the Han population in western China. *PNPLA3* rs738409 was associated with the AST level in MAFLD patients. *GCKR* variants were associated with increased TG levels and reduced serum fasting glucose in patients with MAFLD. In addition, there is no synergistic SNP–SNP interaction between *PNPLA3 rs738409* and other SNPs studied in our research on MAFLD.

## FUNDING INFORMATION

This work was supported by the National Natural Science Foundation of China (No. 81902142) and the Science & Technology Department of Sichuan Province (No. 2018SZ0382 and No. 2019HXFH052).

## CONFLICT OF INTEREST

The authors declare no conflicts of interest.

## Supporting information


Table S1
Table S2
Table S3

## Data Availability

The data that support the findings of the current study are available from the corresponding author upon reasonable request.
