# Supplementary material for: Genetic variants associated with metabolic dysfunction‐associated fatty liver disease in western China
Source: J Clin Lab Anal. 2022 Jul 26;36(9):e24626. doi: 10.1002/jcla.24626 (PMC9459258; doi:10.1002/jcla.24626)
Supplement: Supplementary file 1 — Table S1 Table S2 Table S3 [file JCLA-36-e24626-s001.docx]

**Supplement tables**

**Table S1 The sequences of PCR primers.**

| Name | Upper Primer sequence(5’- 3’) | Lower Primer sequence(5’- 3’) | PCR length |
| --- | --- | --- | --- |
| rs1260326 | GTGGAGCAGGTGAAAGAGAAG | GAGGCATCTCCAGGAAGGC | 139 |
| rs4808199 | TAGACCTTGATCCTGCAGCAG | CCCATTCAAGCTGAGAAACCC | 139 |
| rs58542926 | CAGCCCAGCATGGCACC | GGGTGACAGAGCAAGACTCTG | 169 |
| rs641738 | GACTCCCACCTAGCCTGAAG | GGAAGAAAACCCGACCTTGG | 133 |
| rs738409 | AATTAAAAGGGTGCTCTCGCC | GAAAGCCGACTTACCACGC | 130 |
| rs780094 | CAGGTGATCCCCCAACCTTG | CTTATTCTGCTCCAGGGCCC | 139 |

**Table S2 The information of the LDR reaction probes.**

| Probe name | Probe sequence(5’-3’) | LDR length |
| --- | --- | --- |
| rs58542926R_modify | P-GGAGCTGTATTTGCCTTTTCCATTTTTTTTTTTTTTTTTTTTTT-FAM |  |
| rs58542926R_G | TTTTTTTTTTTTTTTTTTTTTTAGGAAGAAGGCAGGCCTGATCTC | 89 |
| rs58542926R_A | TTTTTTTTTTTTTTTTTTTTTTTTAGGAAGAAGGCAGGCCTGATCTT | 91 |
| rs780094_modify | P-TTTGCTGATCAATACATTTGTTTTTTTTTTTTTTTTTTTTTTTTTT-FAM |  |
| rs780094_T | TTTTTTTTTTTTTTTTTTTTTTTTTTTTAGACCATGACTGACACATA | 93 |
| rs780094_C | TTTTTTTTTTTTTTTTTTTTTTTTTTTTTTAGACCATGACTGACACATG | 95 |
| rs1260326_modify | P-GCAAGGTCTGACCCACGGTGTTTTTTTTTTTTTTTTTTTTTTTTTTTT-FAM |  |
| rs1260326_C | TTTTTTTTTTTTTTTTTTTTTTTTTTTTGTCACGGCTGGACTCTCACCG | 97 |
| rs1260326_T | TTTTTTTTTTTTTTTTTTTTTTTTTTTTTTGTCACGGCTGGACTCTCACCA | 99 |
| rs641738_modify | P-GTGGCTGGCCCCCCGGGAGGTTTTTTTTTTTTTTTTTTTTTTTTTTTTTTTT-FAM |  |
| rs641738_C | TTTTTTTTTTTTTTTTTTTTTTTTTTTTTTGAAGCCTGGGGCTCCTCTAGGGG | 105 |
| rs641738_T | TTTTTTTTTTTTTTTTTTTTTTTTTTTTTTTTGAAGCCTGGGGCTCCTCTAGGGA | 107 |
| rs738409_modify | P-ATGAAGCAGGAACATACCAATTTTTTTTTTTTTTTTTTTTTTTTTTTTTTTTTTTT-FAM |  |
| rs738409_G | TTTTTTTTTTTTTTTTTTTTTTTTTTTTTTTTTTGGATAAGGCCACTGTAGAAGGGC | 113 |
| rs738409_C | TTTTTTTTTTTTTTTTTTTTTTTTTTTTTTTTTTTTGGATAAGGCCACTGTAGAAGGGG | 115 |
| rs4808199_modify | P-CTCTCTGCCCCGAGGGTGGGTTTTTTTTTTTTTTTTTTTTTTTTTTTTTT-FAM |  |
| rs4808199_G | TTTTTTTTTTTTTTTTTTTTTTTTTTTTTTTTGTATCTGCCAATCCAAGAC | 101 |
| rs4808199_A | TTTTTTTTTTTTTTTTTTTTTTTTTTTTTTTTTTGTATCTGCCAATCCAAGAT | 103 |

**Table S3 The summary of** **SNP-SNP interactions detected by logistic regression.** **Logistic regression analysis was adjusted by sex, age, BMI, corresponding SNPs.**

| SNP 1 | Gene | SNP 2 | Gene | *P* | *Adjust P* ^†^ | OR (95% CI) |
| --- | --- | --- | --- | --- | --- | --- |
| rs738409 | *PNPLA3* | rs58542926 | *TM6SF2* | 0.944 | 0.990 | 1.032(0.426~2.593) |
| rs738409 | *PNPLA3* | rs780094 | *GCKR* | 0.199 | 0.426 | 0.765(0.507~1.149) |
| rs738409 | *PNPLA3* | rs1260326 | *GCKR* | 0.188 | 0.426 | 0.761(0.505~1.140) |
| rs738409 | *PNPLA3* | rs641738 | *MBOAT7* | 0.190 | 0.426 | 1.369(0.859~2.199) |
| rs738409 | *PNPLA3* | rs4808199 | *GATAD2A* | 0.527 | 0.945 | 0.858(0.534~1.382) |
| rs58542926 | *TM6SF2* | rs780094 | *GCKR* | 0.990 | 0.990 | 0.994(0.383~2.586) |
| rs58542926 | *TM6SF2* | rs1260326 | *GCKR* | 0.942 | 0.990 | 0.966(0.38~2.422) |
| rs58542926 | *TM6SF2* | rs641738 | *MBOAT7* | 0.677 | 0.945 | 0.8(0.275~2.299) |
| rs58542926 | *TM6SF2* | rs4808199 | *GATAD2A* | 0.084 | 0.420 | 2.659(0.915~8.892) |
| rs780094 | *GCKR* | rs1260326 | *GCKR* | 0.693 | 0.945 | 0.923(0.619~1.376) |
| rs780094 | *GCKR* | rs641738 | *MBOAT7* | 0.596 | 0.945 | 0.885(0.561~1.389) |
| rs780094 | *GCKR* | rs4808199 | *GATAD2A* | 0.028 | 0.210 | 0.591(0.367~0.943) |
| rs1260326 | *GCKR* | rs641738 | *MBOAT7* | 0.890 | 0.990 | 0.969(0.617~1.517) |
| rs1260326 | *GCKR* | rs4808199 | *GATAD2A* | 0.015 | 0.210 | 0.561(0.35~0.891) |
| rs641738 | *MBOAT7* | rs4808199 | *GATAD2A* | 0.177 | 0.426 | 1.437(0.855~2.465) |

^†^  Adjust *p* value was adjusted by Benjamini-Hochberg.
